# Damage control surgery and open abdomen in Hinchey III–IV acute diverticulitis: current practice from a nationwide Italian survey

**DOI:** 10.1007/s13304-026-02834-0

**Published:** 2026-09-10

**Authors:** Alessio Giordano, Manuela Mastronardi, Andrea Mingoli, Hayato Kurihara, Eugenio Cucinotta, Mauro Zago, Massimo Chiarugi, Roberto Cirocchi, Paolo Prosperi, Alan Biloslavo, Gianluca Costa, DCS Collaborative group, Pietro Fransvea, Valerio Cozza, Dario Tartaglia, Fabio Rondelli, Simone Squillante, Antonio Brillantino, Domenico Spoletini, Giovanni Maria Garbarino, Simonetta Iacovitti, Valentina Miacci, Giovanni Giglielmo Iaracca, Filippo Carannante, Ilaria Martire, Federico Tomassini, Diletta Cassini, Barbara Frezza, Marco De Prizio, Chiara Miligi, Giovanni Bellanova, Pietro Anoldo, Giuseppe Brisinda, Gabriele Bellio, Marco Ceresoli, Massimiliano Ardu, Stefano Ferfoglia, Tommaso Fontana, Pietro Bisagni, Marcello Della Corte, Paola Addariochieco, Anna Angrisano, Michele Benedetti, Paola Fugazzola, Massimo Antropoli, Stefano Cioffi, Bruno Cirillo, Christian Galatioto, Maximilian Scheiterle, Luca Ferrario, Corrado Rispoli, Giovanni Casciaro, Fausto Rosa, Luca Ghirardelli, Luca Ghirardelli, Stefano Magnone, Alessandra Lucchetta, Piero Venezia, Martina Ceolin, Simone Sebastiani, Carlo Bergamini, Fabrizio Sammartano, Miriam Vitrano, Claudia Zaghi, Giulio Iacob, Federico Coccolini, Nicolo Mariani, Andrea Martina Guida, Andrea Divizia, Sabina Magalini, Stefano Valente, Paolo Beltempo, Gianluca Liotta, Emilio Gentile Warschauer, Valerio Cozza, Valentina Di Paola, Chiara Cipressi, Simone Targa, Mauro Montuori, Giovanni Battista Damiani, Gabriele Pozzo, Mauro Santarelli, Luca Lepre, Alessia Malagnino, Federico Mariani, Livio Mortati, Kurihara Hayato, Leonardo Delogu, Savino Occhionorelli, Gianluca Baiocchi, Genoveffa Balducci, Daniele Delogu, Paolo Mercantini, Giuseppe Evola, Camilla Cremonini, Diego Mariani, Antonio La Greca, Luigi Cobuccio, Silvia Strambi, Dario Iadicola, Simona Meneghini, Guido Basile, Desire Pantalone, Antonio Crucitti, Antonello Mirabella, Graziano Pernazza, Jacopo Mazzetti, Fabio Pio Curci, Massimiliano Di Paola, Stefano Rossi, Laura Antolino, Edelweiss Licitra, Roberta Tutino, Mauro Podda, Monica Ortenzi, Anna Guariniello, Marco Caricato, Domenico Marchi, Mario Sorrentino, Giuditta Morfino, Francesco Fleres, Sondra Schmitz, Vincenzo Schiavone, Teresa Sinicropi, Pasquale Avella, Pierpaolo Castagiuolo

**Affiliations:** 1 Department of Emergency and Acceptance, Emergency Surgery Unit, Careggi University Hospital, Florence, Italy; 2 Department of Medicine, Surgery and Health Sciences, University of Trieste, Strada di Fiume 447, 34149 Trieste, TS Italy; 3 Department of Surgery, Sapienza University of Rome, Rome, Italy; 4 Department of Emergency Surgery, Fondazione IRCCS Ca’ Granda Ospedale Maggiore Policlinico, Milan, Italy; 5 Department of Human Pathology of the Adult and Evolutive Age “Gaetano Barresi,” Section of General Surgery, University of Messina, Messina, Italy; 6 General Surgery Department, Lecco Hospital, Lecco, Italy; 7 Emergency and General Surgery Unit, New Santa Chiara University Hospital, University of Pisa, Pisa, Italy; 8 Department of Medicine and Surgery, University of Perugia, Perugia, Italy; 9 Department of Life Sciences, Health and Health Professions, Link Campus University, Rome, Italy

**Keywords:** Acute diverticulitis, Damage control surgery, Open abdomen, Hartmann's procedure, Peritonitis, Survey

## Abstract

**Introduction:**

Damage control surgery (DCS) with staged reconstruction may offer an alternative to Hartmann’s procedure in selected unstable patients with generalized peritonitis from acute diverticulitis, but its adoption in routine practice is uncertain.

**Methods:**

A nationwide web-based survey explored management of Hinchey III-IV acute diverticulitis, institutional use of open abdomen, and knowledge and application of DCS. Categorical data were summarized as n (%). The survey was endorsed by Italian Society of Emergency and Trauma Surgery (SICUT).

**Results:**

Among 175 respondents, 71.4% reported using Hartmann’s procedure in at least half of severe cases, whereas 37.7% used primary anastomosis in at least one-quarter. Open abdomen was available in 82.3% of institutions. Awareness of DCS was reported by 148/174 (85.1%), and 116/168 (69.0%) had used it for severe diverticulitis; however, only 33/147 (22.4%) used a clinical score. Hemodynamic instability (83.1%), severe peritoneal contamination (73.2%), and the opportunity for delayed anastomosis (64.1%) were the main indications. Respondents with greater emergency surgery commitment more often reported primary anastomosis (48.1% vs. 20.9%; *P* < 0.001), open abdomen availability (93.5% vs. 64.2%; *P* < 0.001), and previous DCS use (75.5% vs. 58.1%; *P* = 0.024).

**Conclusion:**

DCS is familiar to and has been used by most respondents, but selection and implementation remain heterogeneous and rarely score-based. Standardized criteria, shared terminology, and prospective outcome evaluation are needed.

**Supplementary Information:**

The online version contains supplementary material available at https://doi.org/10.1007/s13304-026-02834-0.

## Introduction

Perforated acute colonic diverticulitis with generalized purulent or fecal peritonitis remains a life-threatening emergency. For Hinchey III-IV disease, operative decision-making must reconcile rapid source control with the risks of anastomotic failure, permanent stoma, and the physiological burden of a definitive procedure in a septic or unstable patient [1–4]. Although Hartmann’s procedure has historically been the dominant strategy, contemporary trials and guidelines support resection with primary anastomosis, with or without diversion, in selected stable and immunocompetent patients [4–7].

Damage control surgery (DCS) offers a different pathway for patients in whom immediate definitive reconstruction may be unsafe. The initial operation is abbreviated to achieve source control, typically through resection of the diseased segment, lavage, temporary control of the bowel ends, and temporary abdominal closure. Following resuscitation, a planned second-look operation permits reassessment and either delayed anastomosis or end colostomy [8–12]. This approach may reduce permanent stoma formation while transferring the most complex reconstructive decision to a physiologically optimized setting.

However, the supporting evidence remains limited and heterogeneous. WSES guidance suggests staged DCS in selected unstable patients with diffuse peritonitis, while emphasizing that open abdomen (OA) should not be used indiscriminately because it carries risks of enteroatmospheric fistula, frozen abdomen, fluid and protein loss, and failure of fascial closure [1, 13, 14]. Randomized and observational studies have reported encouraging continuity and stoma outcomes, but selection criteria, first-stage techniques, temporary abdominal closure methods, and timing of reconstruction vary considerably [8–15].

Understanding how surgeons currently interpret and apply these concepts is therefore essential before protocols or comparative trials can be designed. This survey aimed to describe contemporary practice for severe complicated diverticulitis, quantify awareness and previous use of DCS and OA, and explore whether practice differed according to the proportion of professional activity devoted to emergency surgery.

## Methods

### Study design and participants

A cross-sectional, web-based survey was conducted among surgeons involved in emergency and general surgery in Italy. The survey was available from [INSERT START DATE] to [INSERT END DATE] and was disseminated through [INSERT SCIENTIFIC SOCIETIES, MAILING LISTS, AND/OR PROFESSIONAL NETWORKS]. Participation was voluntary and responses were collected without patient-level data. The report was prepared with reference to the Checklist for Reporting Results of Internet E-Surveys (CHERRIES) [16]. The number of surgeons reached through all distribution channels was not available; therefore, a conventional response rate could not be calculated. The survey was endorsed by Italian Society of Emergency and Trauma Surgery (SICUT).

### Questionnaire and outcomes

The structured Italian-language questionnaire comprised 25 items. The first section recorded respondent age, hospital type and size, proportion of monthly activity devoted to emergency surgery, institutional annual volume of emergency operations for Hinchey III-IV diverticulitis, and the typical age of operated patients. The second section examined the estimated institutional use of primary anastomosis, protective ileostomy, proximal colonic lavage, Hartmann’s procedure, and laparoscopy. The third section addressed institutional OA practice, annual OA volume across all indications, temporary abdominal closure techniques, awareness and previous use of DCS for severe diverticulitis, reported DCS volumes, reasons for selecting DCS with OA, use of clinical scores, and postoperative location. (Supplementary material 1)

The principal descriptive outcomes were awareness of DCS, previous DCS use for severe diverticulitis, institutional OA availability, reported operative strategy, reasons for DCS selection, and use of a clinical score. Multiple-response items were analyzed by counting each option independently; consequently, percentages may sum to more than 100%. Free-text responses were retained for qualitative review but were not used for formal inferential comparisons.

### Statistical analysis

All 175 records in the supplied survey dataset were included. Categorical variables are presented as counts and percentages using the number of available responses to each item as the denominator; missing responses were not imputed. For an exploratory comparison, respondents were grouped according to whether < 30% or ≥ 30% of their monthly working time was devoted to emergency surgery. Binary outcomes were compared using two-sided Fisher’s exact tests. Because these analyses were exploratory, no adjustment was made for multiple testing. A two-sided P value < 0.05 was considered statistically significant. Analyses were performed using Python 3.12, pandas 2.2.3, and SciPy 1.17.0.

### Ethics

Due to the nature of the study, ethics committee approval was not needed.

## Results

### Respondent and institutional characteristics

The analysis included 175 respondents. The largest age group was 31–40 years (65/175, 37.1%). More than half worked in a level II hospital (97/175, 55.4%), and 82/175 (46.9%) worked in hospitals with more than 400 beds. Overall, 108/175 (61.7%) devoted at least 30% of their monthly activity to emergency surgery. Sixty-five respondents (37.1%) reported at least 30 emergency operations for Hinchey III-IV diverticulitis per year at their institution (Table 1).

**Table 1:** Respondent and institutional characteristics (*N* = 175)

Characteristic	*n* (%)
*Respondent age, years*	
< 30	7 (4.0%)
31–40	65 (37.1%)
41–50	40 (22.9%)
51–60	36 (20.6%)
> 60	27 (15.4%)
*Hospital type*	
Basic hospital	13 (7.4%)
Level I hospital	57 (32.6%)
Level II hospital	97 (55.4%)
Other emergency surgery facility	8 (4.6%)
*Hospital beds*	
< 200	31 (17.7%)
200–400	62 (35.4%)
> 400	82 (46.9%)
*Monthly working time devoted to emergency surgery*	
< 20%	26 (14.9%)
20–30%	41 (23.4%)
30–50%	45 (25.7%)
> 50%	63 (36.0%)
*Annual emergency operations for Hinchey *III-IV* diverticulitis*	
< 10	14 (8.0%)
10–20	46 (26.3%)
20–30	50 (28.6%)
30–40	28 (16.0%)
40–50	23 (13.1%)
> 50	14 (8.0%)
*Typical age of operated patients, years*	
30–50	3 (1.7%)
50–70	122 (69.7%)
70–80	47 (26.9%)
> 80	3 (1.7%)

Percentages use the number of available responses as the denominator. All variables in this table had 175 responses

### Operative strategies for severe diverticulitis

**Fig. 1:**
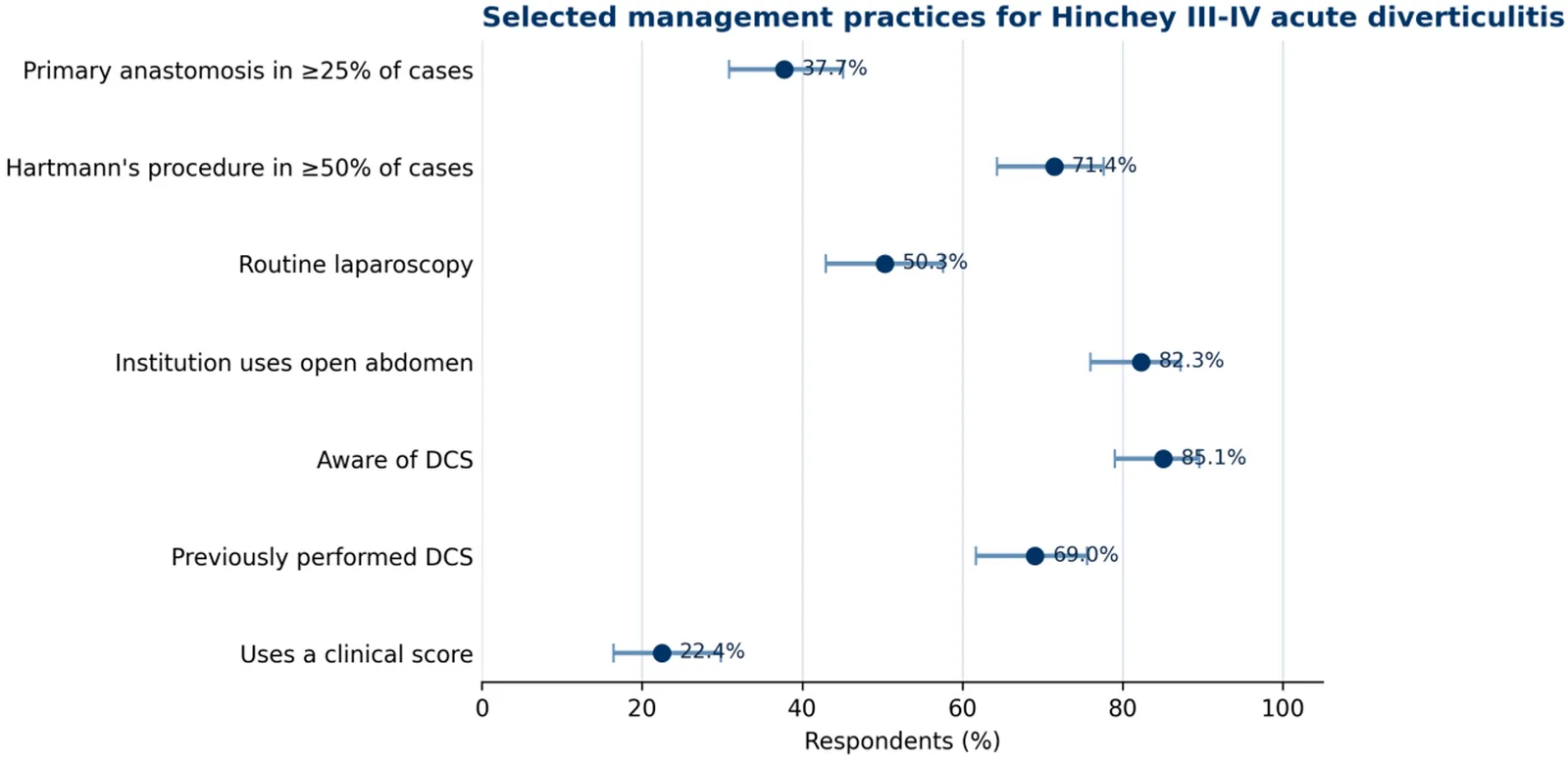
Selected management practices for Hinchey III-IV acute diverticulitis. Points indicate observed proportions and whiskers indicate 95% Wilson confidence intervals. Denominators are the available responses for each item

Hartmann’s procedure remained the predominant reported strategy: 125/175 (71.4%) estimated that it was performed in at least half of Hinchey III-IV cases. Conversely, 109/175 (62.3%) reported primary anastomosis in fewer than one-quarter of cases, and only 66/175 (37.7%) used it in at least one-quarter. When primary anastomosis was performed, 105/174 (60.3%) usually created a protective ileostomy, whereas proximal colonic lavage was routinely used by 40/173 (23.1%). A laparoscopic approach was routinely attempted unless contraindicated by 88/175 (50.3%), used only for diagnostic uncertainty by 65/175 (37.1%), and never used by 22/175 (12.6%) (Table 2 and Fig. 1).

**Table 2:** Reported operative strategies for Hinchey III-IV acute diverticulitis

Characteristic	*n* (%)
*Proportion treated by resection and primary anastomosis*	
< 25%	109 (62.3%)
25–50%	51 (29.1%)
50–75%	11 (6.3%)
> 75%	4 (2.3%)
*Protective ileostomy usually created after primary anastomosis*	
Yes	105 (60.3%)
No	69 (39.7%)
*Proximal colonic lavage usually performed*	
Yes	40 (23.1%)
No	133 (76.9%)
*Proportion treated by Hartmann’s procedure*	
< 25%	8 (4.6%)
25–50%	41 (23.4%)
50–75%	64 (36.6%)
> 75%	61 (34.9%)
*Use of laparoscopy*	
Routine laparoscopic approach unless contraindicated	88 (50.3%)
Laparoscopy only for diagnostic uncertainty	65 (37.1%)
Never	22 (12.6%)

Percentages use available responses as the denominator. Denominators were 175 except protective ileostomy (*n* = 174) and proximal colonic lavage (*n* = 173)

### Open abdomen and damage control surgery

**Fig. 2:**
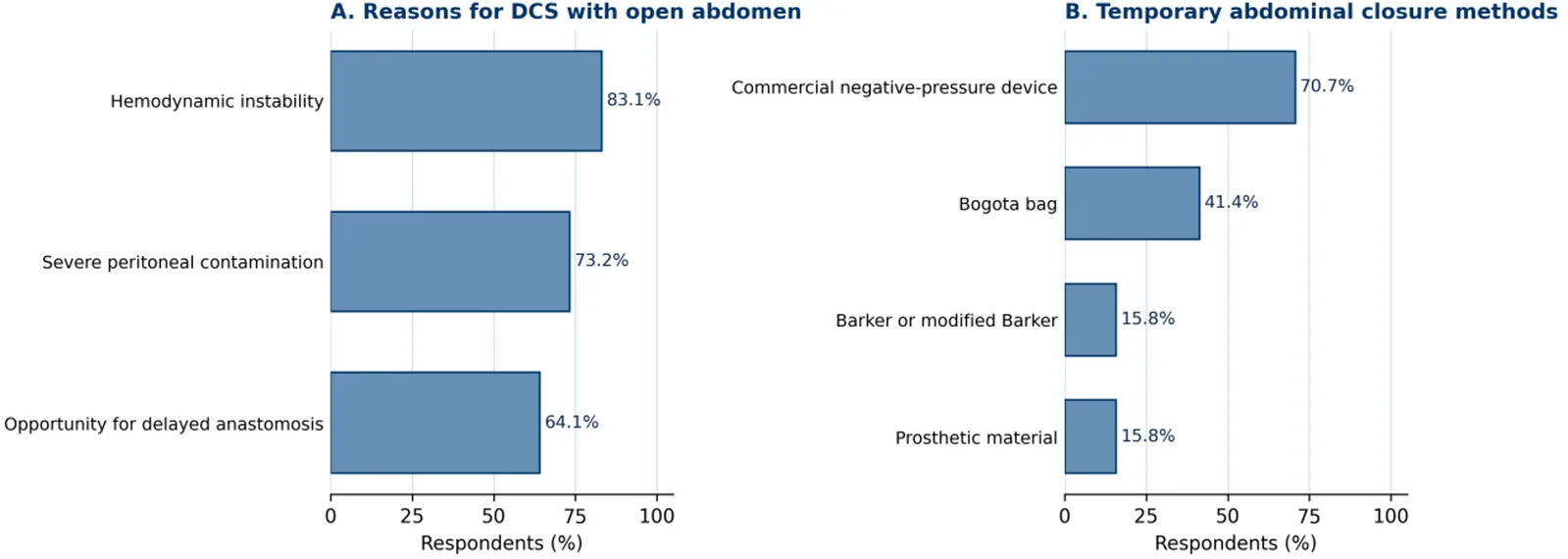
Reported reasons for selecting damage control surgery with open abdomen (**A**) and temporary abdominal closure methods (**B**). Items allowed multiple responses; percentages therefore do not sum to 100%. DCS, damage control surgery

Institutional use of OA for any indication was reported by 144/175 respondents (82.3%). Among 161 respondents providing annual volume, 68 (42.2%) reported more than 10 OA cases per year. Commercial negative-pressure systems were the most common temporary abdominal closure method (94/133, 70.7%), followed by the Bogota bag (55/133, 41.4%); Barker or modified Barker techniques and prosthetic material were each reported by 21/133 (15.8%) (Table 4 and Fig. 2).

**Table 3:** Open abdomen and damage control surgery practice

Characteristic	*n* (%)
*Open abdomen practice*	
Open abdomen used at the institution	144 (82.3%)
*Annual open abdomen case volume (all indications)*	
< 5	51 (31.7%)
6–10	42 (26.1%)
10–30	39 (24.2%)
> 30	29 (18.0%)
Commercial negative-pressure device	94 (70.7%)
Bogota bag	55 (41.4%)
Barker or modified Barker technique	21 (15.8%)
Prosthetic material	21 (15.8%)
*Damage control surgery*	
Aware of DCS for severe acute diverticulitis	148 (85.1%)
Previously performed DCS for severe acute diverticulitis	116 (69.0%)
*Annual lavage-and-drainage-only procedures among DCS users*	
0	44 (38.3%)
< 10	66 (57.4%)
> 10	5 (4.3%)
*Annual resection-plus-open-abdomen procedures among DCS users*	
0	17 (14.9%)
< 10	82 (71.9%)
> 10	15 (13.2%)
Hemodynamic instability	118 (83.1%)
Severe peritoneal contamination	104 (73.2%)
Opportunity for delayed anastomosis	91 (64.1%)
Uses a clinical score to guide the decision	33 (22.4%)
Routine postoperative ICU admission	92 (65.2%)

Percentages use available responses as the denominator. Temporary abdominal closure methods and DCS indications allowed multiple responses, so percentages may exceed 100% when summed. DCS, damage control surgery; ICU, intensive care unit

Awareness of DCS for severe acute diverticulitis was reported by 148/174 respondents (85.1%), and 116/168 (69.0%) had previously used DCS in this setting. Among previous DCS users with available data, 97/114 (85.1%) reported at least one annual resection-plus-OA procedure, although 82/114 (71.9%) performed fewer than 10 per year. Lavage-and-drainage-only procedures were also reported by 71/115 (61.7%) of DCS users, suggesting heterogeneity in how the damage-control label was applied.

The leading reasons for choosing DCS with OA were hemodynamic instability (118/142, 83.1%), severe peritoneal contamination (104/142, 73.2%), and the opportunity to perform an anastomosis at a later stage (91/142, 64.1%). Only 33/147 respondents (22.4%) used a clinical score to guide selection. Routine postoperative intensive care admission was reported by 92/141 (65.2%) (Table 4 and Fig. 2).

### Association with emergency surgery commitment

Compared with respondents devoting less than 30% of their activity to emergency surgery (*n* = 67), those with greater commitment (*n* = 108) more often worked in settings performing at least 30 severe diverticulitis operations annually (47.2% vs. 20.9%; *P* < 0.001). They more frequently reported primary anastomosis in at least one-quarter of cases (48.1% vs. 20.9%; *P* < 0.001) and less frequently reported routine protective ileostomy when an anastomosis was created (51.9% vs. 74.2%; *P* = 0.004). OA availability (93.5% vs. 64.2%; *P* < 0.001), DCS awareness (90.7% vs. 75.8%; *P* = 0.009), and previous DCS use (75.5% vs. 58.1%; *P* = 0.024) were also higher. Use of clinical scores remained low and similar between groups (23.7% vs. 20.0%; *P* = 0.680) (Table 3).

**Table 4:** Practices according to the proportion of monthly working time devoted to emergency surgery

Practice	< 30% emergency surgery (*n* = 67)	≥ 30% emergency surgery (*n* = 108)	*P* value
At least 30 annual Hinchey III-IV operations	14 (20.9%)	51 (47.2%)	< 0.001
Primary anastomosis used in at least 25% of cases	14 (20.9%)	52 (48.1%)	< 0.001
Protective ileostomy usually created	49 (74.2%)	56 (51.9%)	0.004
Proximal colonic lavage usually performed	19 (28.8%)	21 (19.6%)	0.195
Hartmann’s procedure used in at least 50% of cases	53 (79.1%)	72 (66.7%)	0.087
Routine laparoscopic approach unless contraindicated	37 (55.2%)	51 (47.2%)	0.352
Open abdomen used at the institution	43 (64.2%)	101 (93.5%)	< 0.001
Aware of DCS for severe acute diverticulitis	50 (75.8%)	98 (90.7%)	0.009
Previously performed DCS for severe acute diverticulitis	36 (58.1%)	80 (75.5%)	0.024
Uses a clinical score to guide the decision	10 (20.0%)	23 (23.7%)	0.680

Values are n (%) among available responses. P values were calculated using two-sided Fisher’s exact tests. The comparisons were exploratory and were not adjusted for multiple testing. DCS, damage control surgery

## Discussion

This survey provides a broad snapshot of current attitudes toward operative management of Hinchey III-IV diverticulitis. Three findings are particularly relevant. First, Hartmann’s procedure remained dominant despite increasing evidence supporting restorative strategies in selected patients. Second, both OA capability and familiarity with DCS were widespread, and more than two-thirds of respondents had used DCS for severe diverticulitis. Third, implementation was poorly standardized: clinical scores were seldom used, reported procedure definitions varied, and practice differed substantially according to the respondent’s exposure to emergency surgery.

The predominance of Hartmann’s procedure is consistent with the persistent caution surrounding emergency anastomosis in generalized peritonitis. However, randomized trials such as DIVERTI and LADIES, together with subsequent meta-analyses, have shown that primary anastomosis can achieve similar short-term morbidity and mortality while improving stoma-free survival in appropriately selected stable patients [5–7]. The present results do not imply that primary anastomosis should be generalized to unstable patients. Rather, the marked preference for Hartmann’s procedure highlights how patient physiology, institutional resources, surgeon experience, and nighttime operating conditions continue to shape practice beyond guideline statements.

The open abdomen (OA) technique is a life-saving option frequently associated with damage control surgery (DCS). While its role in trauma and in abdominal compartment syndrome is well described [17, 18] and represents a keystone, its use in others non-trauma patients is more controversial, especially in secondary peritonitis [19].

The management of abdominal sepsis is complex and multimodal and the standard of care for these patients is in continuous evolution, moreover, this condition is often associated with multiple variables like physiologic derangements or inadequate source control which makes the management even more challenging. The use of OA in non-trauma patients is theorized in WSES guidelines, as previously mentioned [13], under some circumstances: physiological derangement, the need for a deferred intestinal anastomosis, visceral oedema with the risk of ACS and a failure to source control which involves a persistent source of infection. However, these are low grade of recommendation (Grade 2 C) and there are no definitive data regarding an improvement in survival connected to the use of OA in this scenario. From the listed indications to perform OA in peritonitis, we dwelled on “failure to source control” which in our opinion resulted the more specific in an abdominal sepsis scenario. Although the effort to clearly define the “adequate source control” in which OA may be used [20, 21], in clinical practice the lack of definitive guidelines could expose to a misuse of this strategy.

DCS may bridge the gap between immediate primary anastomosis and definitive end colostomy. By separating source control from reconstruction, it allows correction of shock, acidosis, hypothermia, coagulopathy, and organ dysfunction before the continuity decision. Published series and meta-analyses have reported gastrointestinal continuity rates around 60% or higher with acceptable mortality, although selection bias and heterogeneous inclusion criteria remain important concerns [8–12, 22]. A recent study [19] conducted at a high-volume center on the general treatment of secondary peritonitis demonstrated that approximately 10.5% of the total OA performed allowed a reduction in the percentage of stomas and the creation of an anastomosis during the second look, specifically in the group of severe diverticulitis. These findings are encouraging, but DCS should remain a selective strategy rather than a default response to all generalized peritonitis.

The current survey reveals a possible terminology problem. Most previous DCS users reported resection with OA, which is consistent with the staged source-control concept. Nevertheless, a substantial proportion also classified lavage and drainage alone as DCS. Laparoscopic lavage may have a role in selected purulent peritonitis, but it is conceptually distinct from an abbreviated resection followed by planned reconstruction. Without agreed definitions, comparisons between centers and interpretation of future outcome studies will remain difficult. Consensus documents should therefore specify the essential components of a diverticulitis DCS pathway: patient selection, initial source-control steps, management of bowel ends, temporary abdominal closure, resuscitation targets, timing of second look, and criteria for anastomosis versus stoma.

The high prevalence of institutional OA use indicates that the necessary capability is widely available, but OA itself is not a benign treatment. WSES recommendations favor negative-pressure wound therapy with fascial traction and advocate closure as soon as the patient can physiologically tolerate it [13, 14]. Commercial negative-pressure systems were the most frequently reported method in this survey, which broadly aligns with these recommendations. Nonetheless, the continuing use of non-negative-pressure techniques and the variation in postoperative destination underline the need for local protocols involving emergency surgeons, intensivists, anesthesiologists, and abdominal wall expertise [23]. 

Greater emergency surgery commitment was associated with higher reported institutional volume, greater adoption of primary anastomosis, wider OA availability, and more frequent DCS experience. These associations are plausible: repeated exposure may improve selection, technical familiarity, and access to multidisciplinary pathways. They should not be interpreted causally, because respondents with high emergency commitment were also more likely to work in high-volume environments. The result nevertheless suggests that dissemination of DCS cannot rely on written guidance alone; structured training, simulation of staged decision-making, and referral or proctoring networks may be required for lower-volume hospitals.

The low use of clinical scores is a central implementation gap. No existing score can replace intraoperative judgment, and the evidence does not support a single mandatory threshold. Even so, standardized documentation of physiology - including vasopressor requirement, lactate, organ dysfunction, frailty, comorbidity, and contamination - would make selection more transparent and auditable. A pragmatic algorithm combining physiological instability, source-control complexity, and feasibility of delayed reconstruction may be more useful than attempting to identify one universal score.

This study had several limitations. Recruitment used professional distribution channels and the number of surgeons reached was unavailable, preventing calculation of a response rate and introducing selection bias. Responses were self-reported estimates rather than audited operative data, and multiple surgeons may have represented the same institution. Several conditional questions had missing responses, so available-case denominators varied. The questionnaire did not capture patient-level physiology, operative details, timing of reconstruction, fascial closure, or clinical outcomes. The dichotomization of emergency surgery commitment at 30% and the associated comparisons were exploratory, with no correction for multiple testing. Finally, the survey wording allowed variation in the interpretation of DCS, which is both a limitation and an important finding. These factors limit generalizability and preclude conclusions about comparative effectiveness.

## Conclusions

Among respondents to this Italian survey, Hartmann’s procedure remained the dominant operation for severe acute diverticulitis, while OA and DCS were widely known and frequently used. The contrast between high DCS adoption and low score-based selection, together with variation in reported techniques, indicates a need for clearer definitions and standardized multidisciplinary pathways. Prospective registries and pragmatic comparative studies should evaluate not only mortality and morbidity, but also fascial closure, anastomotic outcomes, stoma-free survival, resource use, and patient-reported outcomes.

## Supplementary Information

Below is the link to the electronic supplementary material.

Supplementary Material 1.
