# Supplementary material for: Damage control surgery and open abdomen in Hinchey III–IV acute diverticulitis: current practice from a nationwide Italian survey
Source: Updates Surg. 2026 Sep 10;78(7):2885–92. doi: 10.1007/s13304-026-02834-0 (PMC13751567; doi:10.1007/s13304-026-02834-0)
Supplement: Supplementary file 1 [file 13304_2026_2834_MOESM1_ESM.docx]

**SURVEY STRUCTURE**

**Informed Consent**

I hereby declare that I have been informed, before participating in the survey, of the right to stop participating at any time, without providing any reason, without any penalty and obtaining the non-use of the data provided by me. I also declare that I have been informed of the purpose of this survey and that the data will remain confidential and protected according to the Personal Data Protection Code (Legislative Decree no. 196/2003). I am aware of the fact that only the people conducting the research will have access to the data, limited to the purposes of their processing and the anonymous publication of the results for scientific purposes. For further information I can contact the research managers. (I agree/ I do not agree)

**Module 1**

**E-mail address ***

________________________________________________________________________________________

**1. What is your age? ***

○ <30

○ 31–40

○ 41–50

○ 51–60

○ >60

**2. What type of hospital do you work in? ***

○ Basic hospital (Emergency Department with a limited number of specialties and an on-call support network)

○ Level I hospital / first-level Emergency Department (several specialties with active on-call medical coverage; 24/7 access to radiology with CT and ultrasound, laboratory, and transfusion services)

○ Level II hospital / second-level Emergency Department (all major specialties involved in traumatic and non-traumatic emergencies, mostly with 24/7 in-house coverage; including university hospitals and research hospitals)

○ Other type of hospital providing emergency surgery for patients admitted through the Emergency Department

**3. How many beds does your hospital have?**

○ <200

○ 200–400

○ >400

**4. What percentage of your monthly working time is dedicated to emergency surgery? ***

○ <20%

○ 20–30%

○ 30–50%

○ >50%

**5. How many emergency surgical procedures for severe complicated acute diverticulitis (Hinchey–Wasvary III–IV) are performed at your institution each year? ***

○ <10

○ 10–20

○ 20–30

○ 30–40

○ 40–50

○ >50

**6. What is the mean age of patients with acute diverticulitis undergoing emergency surgery at your institution?**

○ <30

○ 30–50

○ 50–70

○ 70–80

○ >80

**7. In severe complicated acute diverticulitis (Hinchey–Wasvary III–IV), in what percentage of cases is primary resection and anastomosis performed as an emergency procedure at your institution? ***

○ <25%

○ 25–50%

○ 50–75%

○ >75%

**8. When primary resection and anastomosis is performed in an emergency setting, do you USUALLY protect the anastomosis with a diverting ileostomy?**

○ Yes

○ No

**9. When primary resection and anastomosis is performed in an emergency setting, do you USUALLY perform intraoperative lavage of the proximal colon?**

○ Yes

○ No

**10. In severe complicated acute diverticulitis (Hinchey–Wasvary III–IV), in what percentage of cases is Hartmann's procedure performed at your institution? ***

○ <25%

○ 25–50%

○ 50–75%

○ >75%

**11. In severe complicated acute diverticulitis (Hinchey–Wasvary III–IV), under which of the following conditions do you use a laparoscopic approach? ***

○ Always (unless there are absolute contraindications to laparoscopy)

○ Only in cases of diagnostic uncertainty

○ Never

**12. Is the Open Abdomen technique used at your institution? ***

○ Yes

○ No

**13. If you answered Yes to #12, under which circumstances do you perform an Open Abdomen? (You may list more than one indication.)**

________________________________________________________________________________________

________________________________________________________________________________________

**14. How many patients in total are treated with an Open Abdomen at your institution each year (FOR ANY INDICATION)?**

○ <5

○ 6–10

○ 10–30

○ >30

**15. Which technique(s) do you use for Temporary Abdominal Closure (TAC)? (You may select more than one answer.)**

☐ Commercial negative-pressure therapy devices

☐ Bogotá bag

☐ Barker vacuum pack or modified Barker technique

☐ Prosthetic material

☐ Other

**16. If you answered Other to #15, please specify. (You may list more than one technique.)**

________________________________________________________________________________________

________________________________________________________________________________________

**17. Are you aware of the potential use of Damage Control Surgery (DCS) strategies in severe acute diverticulitis (Hinchey–Wasvary III–IV)? ***

○ Yes

○ No

**Module 2**

**18. Have you ever adopted a Damage Control Surgery procedure in a case of severe acute diverticulitis?**

○ Yes

○ No

**19. Which Damage Control Surgery procedures have you used in diverticulitis? (You may list more than one technique.)**

________________________________________________________________________________________

________________________________________________________________________________________

**20. If you answered Yes to #18, how many procedures consisting ONLY of lavage and drainage for complicated diverticulitis are performed at your institution in one year?**

○ 0

○ <10

○ >10

**21. If you answered Yes to #18, how many DCS procedures involving resection and Open Abdomen for complicated diverticulitis are performed at your institution in one year?**

○ 0

○ <10

○ >10

**22. Why do you choose to perform DCS with Open Abdomen in acute diverticulitis? (You may select more than one answer.)**

☐ Hemodynamic instability

☐ Severe peritoneal contamination (e.g., feculent peritonitis or diffuse pus involving more than one abdominal quadrant)

☐ Possibility of performing the anastomosis during a second-look procedure

☐ Other

**23. Do you use any clinical score to guide your decision?**

○ Yes

○ No

**24. Which score do you use? (You may list more than one.)**

________________________________________________________________________________________

________________________________________________________________________________________

**25. When you perform DCS with Open Abdomen for acute diverticulitis, where is the patient managed postoperatively? (More than one answer is possible.)**

☐ Always in the Intensive Care Unit (ICU)

☐ In the ICU only if hemodynamically unstable (requiring vasopressor infusion)

☐ In the ICU whenever a negative-pressure therapy device is used

☐ In the ICU only when other Temporary Abdominal Closure techniques are used

☐ Other
